# Book Reviews

**Published:** 1873-02-15

**Authors:** 


### Book Reviews.

Hand-Book of Physiology. By William S. Kirkes, M.D. Edited by W. Morrant Baker, F.R., C.S., etc. With two hundred and forty-eight illustrations. Eighth edition. London: John Murray, Albemarle street. Philadelphia: Lindsay & Blakiston. Price $5.00.

A volume of 800 pages, forming a fairly practical, comprehensive *hand-book*, but inferior, in many respects, to our own American text-books on the subject.

Clinical Lectures on Diseases Peculiar to Women. By Lambe Atthill, M.D. University of Dublin. Second edition, revised and enlarged. Philadelphia: Lindsay & Blakiston. Price $2.25.

This is a small volume of 240 pages, illustrated with several lithograph-plates and wood-cuts. It embraces fifteen lectures on the following topics: Leucorrhœa, Amenorrhœa, Dysmenhorrhœa, Menorrhagia, Polypus, Mucous, Cistic, Fibrous, etc.; Fibrous Tumors, Ovarian Cystic Disease; Inflammation of Cervix Uteri; Displacements of Uterus; Enlargements of Uterus; Cancer.

The author lays claim to no special originality in the views put forward on these subjects. He has merely endeavored to present a concise, practical consideration of the recent advances which have been made in the medical and surgical treatment of this class of diseases. The author is a strong advocate of the use of nitric acid as an application to the interior of the uterus, a mode of treatment, he says, "which, I am confident, will yet be practiced as extensively by other obstetric surgeons, as it now is by those of the Dublin school."

Aids to the Diagnosis of Diseases of the Kidneys. By W. R. Basham, M.D. Philadelphia: Lindsay & Blakiston Price $2.

This little volume is mainly made up of lithograph-plates, representing the microscopical appearance of the urinary sediments in the various kidney diseases. The plates are very distinctly and accurately drawn, and, with their aid, the most inexperienced observer should be enabled to distinguish, with tolerable accuracy, the various pathological changes and conditions of the kidneys, as revealed in the microscopical examination of the urine. The text is made up simply of short notes explanatory of the plates.

Obstetric Aphorisms, for the use of Students commencing Midwifery Practice. By Jos. G. Swayne, M.D. Second American, from Fifth English Edition, with Additions, by Edward S. Hutchins, M.D. Philadelphia: Henry C. Lea. For sale by Jansen, McClurg & Co., Chicago.

This little work undertakes merely to supply the student or young practitioner with a few brief and practical directions respecting the management of ordinary labor, and to so far explain the diagnosis of extraordinary cases as to enable them the more easily to decide when they may safely act on their own responsibility, and when they ought to send for assistance.

It is a pocket-companion from which the inexperienced beginner in midwifery practice would frequently derive the greatest comfort and assistance.

Cundurango.—Fluid extract Cundurango, representing 480 grains of the bark in each fluid ounce; also, Cundurango bark, of known reliability. E. H. Sargent, Chemist, 785 Wabash av., Chicago.

Country Practice for Sale.—A desirable country practice for sale. Terms very moderate. For particulars, address S. H. Drake, M.D., Rossville, Allamakee Co., Iowa.

